# New species of *Idris* Förster (Hymenoptera, Platygastroidea) from southeast Asia, parasitoids of the eggs of pholcid spiders (Araneae, Pholcidae)

**DOI:** 10.3897/zookeys.811.29725

**Published:** 2018-12-31

**Authors:** Norman F. Johnson, Huayan Chen, Bernhard A. Huber

**Affiliations:** 1 Department of Evolution, Ecology & Organismal Biology, The Ohio State University, Columbus, OH 43212 USA; 2 Department of Entomology, The Ohio State University, Columbus, OH 43212 USA; 3 Zoological Research Museum Alexander Koenig, Leibniz-Institut für Biodiversität der Tiere, Bonn, Germany

**Keywords:** Araneomorphae, Baeini, Scelioninae, taxonomy, wasps

## Abstract

Four new species of the genus *Idris* Förster (Hymenoptera: Platygastroidea), reared from the eggs of pholcid spiders (Araneae: Pholcidae) in southeast Asia are described on the basis of external morphology and the barcode region of the mitochondrial COI gene. The new species and their hosts are: *I.badius* Johnson & Chen, **sp. n.** (ex *Nipisaphyllicola* (Deeleman-Reinhold), *Panjangehamiguitan* Huber), *I.balteus* Johnson & Chen, **sp. n.** (ex *Panjangecamiguin* Huber), *I.curtus* Johnson & Chen, **sp. n.** (ex *Calapnitanunezae* Huber, *Panjangecamiguin* Huber, *Tissahamiabukittimah* (Huber), *Uthinaluzonica* Simon), and *I.fusciceps* (ex *Belisanakhaosok* Huber).

## Introduction

Egg parasitoid wasps of the tribe Baeini (Hymenoptera: Platygastridae) are one of the major biotic sources of mortality among their spider hosts (Austin 1985), and these wasps are notable both for their abundance and diversity of species. The concept of this tribe has been revised as a consequence of phylogenetic analyses (Carey et al. 2006). There are four core genera that make up the vast majority of species: *Ceratobaeus* Ashmead with 165 described, valid species worldwide; *Idris* Förster with 160 species; *Odontacolus* Kieffer with 55 species; and *Baeus* Haliday with 53 species (Johnson 1992, HOL 2018). While *Odontacolus* appears to be restricted to tropical and subtropical regions, the other genera are widespread throughout the world. Huggert (1979) revised the species of *Idris* and *Ceratobaeus* in the West Palearctic region. This area has a very modest diversity of baeines, only 20 species in total. In contrast, Iqbal and Austin (1997) estimated that in Australia alone the Baeini may total 440 species. Following a focused study of the Australian fauna (Iqbal and Austin 2000) they suggested later that there may be as many as 400 Australian species of *Ceratobaeus* alone. If the genera in tropical Asia, Africa and America are comparably rich, then even a conservative estimate would place the global total in hundreds, if not thousands of species.

The use of the eggs of spiders as hosts among platygastroids is characteristic of baeines, but not limited to them. The genera *Echthrodesis* Masner, *Mirobaeoides* Dodd, and *Neobaeus* Austin also attack these hosts and form a small, separate clade together with *Emibodobia* Ashmead in the analysis of van Noort et al. (2014). *Embidobia* and its close relatives are parasitoids of the eggs of Embiidina (webspinners). Additionally, *Aradophagus* Ashmead has been reported as a spider egg parasitoid (Vetter et al. 2012). According to records summarized in Austin (1985) and Iqbal and Austin (2000) together with those gleaned from specimens in collections, baeines have been reared from 20 families of araneomorph spiders. To date, no baeine has been reared from a host other than a spider. There are no data available to indicate the degree of host specificity within individual wasp species.

The spider hosts in the present paper are all members of Pholcidae, but this is primarily a result of collection trips focused on pholcid spiders and does not imply host specificity of the wasps. Pholcid spider females carry their egg-sacs with their mouthparts until the spiderlings hatch. It is possibly for this direct protection by the female that in most species the eggs are not densely covered by a protective layer of silk but held together by a few turns of silk. This is in contrast to most other spider families where dense and often complex layers of silk are thought to have coevolved with specialized parasitoids and predators (Austin 1985, Hieber 1992). In Pholcidae it seems that few egg parasites have managed to take advantage of the lack of a silk barrier and circumvent the direct protection by the female spider. Only one case has previously been published in Pholcidae (Huber and Wunderlich 2006).

Approximately sixty species of the genera *Idris* and *Ceratobaeus* have been described from tropical Asia, almost entirely from either India or Vietnam (Johnson 1992). Only two species of *Baeus* are known, one from India and the other from Vietnam. There is no comprehensive treatment or identification key for any of the genera, and therefore confident recognition of these species is often next to impossible. The photographic catalogs of primary types of Talamas et al. (2017) and Talamas and Pham (2017) are of limited use in recognition of some of the described species from India and Vietnam respectively. These images are all available at specimage.osu.edu. In most cases, though, the specimens are in such poor condition that positive identification is very difficult. As a result, it is with some trepidation that we offer the description of new species of *Idris* from southeast Asia.

The problem is that we are dealing with (1) a richly speciose genus, (2) a group without any solid, comprehensive treatment for the region, and (3) a group containing numerous described species many of which are more or less unidentifiable. Would the addition of more isolated species descriptions be a contribution toward progress, or would it simply make the problem larger and more intractable?

If the ultimate goal is a complete documentation of the diversity in such a group, then one can imagine various strategies to achieve that aim. The ideal might be a comprehensive monographic treatment based on the totality of specimens existing in collections, the addition of targeted, newly collected material, a review of all existing primary types, and data sets that incorporate as many independent character sources as possible. For many reasons, this standard may be difficult or impossible to achieve, especially when dealing with a genus comprised of hundreds of species. An alternative could be to work gradually toward that same goal by incrementally planting signposts in the terrain, signposts that are well-defined points of reference to guide for future work. This is our goal here: each species description has host records and COI barcode data to supplement the morphological characters.

## Materials and methods

Pholcid spiders were collected manually and egg sacs were checked with a hand-held lens in search of parasitized eggs. Females with parasitized eggs were kept alive in small plastic containers until the wasps emerged. Adult specimens emerged from eight samples. Specimens from these samples were used in both the morphological and molecular analyses:

Mal228: SINGAPORE: Dairy Farm Nature Park (1°21.6'N, 103°46.7'E), 50 m a.s.l., 15.ii.2015 (B.A. Huber, J. Koh). Spider: Uthinaluzonica Simon. 2 specimens sequenced.
Mal256: SINGAPORE: Dairy Farm Nature Park (1°21.6'N, 103°46.7'E), 50 m a.s.l., 15.ii.2015 (B.A. Huber, J. Koh). Spider: Tissahamiabukittimah (Huber). 2 specimens sequenced.
Mal276: MALAYSIA: Perak, Gunung Liang (3°47.7'N, 101°32.0'E), 250 m a.s.l., forest along river, 22.ii.2015 (B.A. Huber, A.R.M. Ghazali, K.A. Braima). Spider: Nipisaphyllicola (Deeleman-Reinhold). 1 specimen sequenced.
Mal305: MALAYSIA: Perak: Gunung Liang (3°47.7'N, 101°32.0'E), 250 m a.s.l., forest along river, 22.ii.2015 (B.A. Huber, A.R.M. Ghazali, K.A. Braima). Spider: Tissahamiagombak (Huber). 2 specimens sequenced.
Mal331: THAILAND: Krabi, ~9 km N Krabi town, degraded forest between plantation and rocks (8°09.9'N, 98°51.7'E), 75 m a.s.l., 7.iii.2015 (B.A. Huber, B. Petcharad). Spider: Belisanakhaosok Huber. 1 specimen sequenced.
Phi291: PHILIPPINES: Bohol, near Loboc, above Loboc River (~9.655N, 124.015E), ~250 m a.s.l., forest near caves, 5.iii.2014 (B.A.Huber). Spider: Panjangecamiguin Huber. 1 specimen sequenced.
PSt1226: PHILIPPINES: Mindanao, Davao Oriental, Mount Hamiguitan Wildlife Sanctuary (access Governor Generoso), site 3 (6.6805N, 126.1591E), 580 m a.s.l., 13.ii.2015 (M.A. Responte). Spider: Panjangehamiguitan Huber. 1 specimen sequenced.
PSt1564: PHILIPPINES: Visayas, Bohol, Bilar, Barangay Riverside, site 5 (9.7052N, 124.1253E), 440 m a.s.l., 15.vi.2015 (M.R.B. Dacar). Spider: Panjangecamiguin Huber. 1 specimen sequenced.

In an additional five samples the parasitoids grew to the pupal stage, but failed to emerge as adults. Pupae from these samples were used in the molecular analyses.

Mal226: SINGAPORE: Upper Selatar Reservoir Park (1°24.0'N, 103°48.4'E), 20 m a.s.l., leaf litter, 15.ii.2015 (B.A. Huber, D. Court). Spider: Uthinaluzonica Simon. 2 specimens sequenced.
Phi271: PHILIPPINES: Mindanao, Mt. Matutum, Kawit Forest, ‘site 1’(6.338N, 125.104E), 950 m a.s.l., along brook, on leaves, 13.ii.2014 (B.A. Huber). Spider: Calapnitanunezae Huber. 2 specimens sequenced.
Phi286: PHILIPPINES: Mindanao, Bukidnon Prov., Santo Domingo (7.782N, 125.397E), 560 m a.s.l., forest remnant along brook, 8–9.ii.2014 (B.A. Huber). Spider: Terangadomingo (Huber). 1 specimen sequenced.
PSt461: PHILIPPINES: Mindanao, Maguindanao, Camp Abubakar (7.5698N, 124.3198E), 14.xii.2014 (N.U. Elias). Spider: Nipisasubphyllicola (Deeleman-Reinhold). 2 specimens sequenced.
PSt84: PHILIPPINES: Mindanao, Marawi City, Mt. Mupo (8.0219N, 124.2986E), 19.xi.2014 (N.U. Elias). Spider: Nipisasubphyllicola (Deeleman-Reinhold). 2 specimens sequenced.

The wasp specimens are deposited in the C.A. Triplehorn Insect Collection, Ohio State University, Columbus, OH (OSUC). The host spiders are deposited at the Zoological Research Museum Alexander Koenig, Bonn, Germany (ZFMK).

Abbreviations and morphological terms used in text: A1, A2, A3: antennomere 1, 2, 3; T1, T2, ... T5: metasomal tergite 1, 2, ... 5. Morphological terminology otherwise generally follows Masner (1980) and Mikó et al. (2007). In the Material Examined section the specimens studied are recorded in an abbreviated format, using unique identifiers (numbers prefixed with “OSUC”) for the individual specimens. The label data for all specimens are recorded in the Hymenoptera Online database, and details on the data associated with these specimens can be accessed at hol.osu.edu by entering the identifier in the form (note the space between the acronym and the number). All new species names have been prospectively registered with Zoobank (Polaszek et al. 2005, www.zoobank.org). The taxonomic descriptions were generated by a database application, vSysLab (vsyslab.osu.edu), designed to facilitate the production of taxon by character data matrices and to integrate those data with the existing taxonomic and specimen-level database. The text output for descriptions is in the format of “Character: Character state (s)”. Polymorphic characters are indicated by semicolon-separated character states. Comparison with holotypes of species described from India and Vietnam were made using the images in Specimage (specimage.osu.edu) referenced in Talamas et al. (2017) and Talamas and Pham (2017). Images and measurements were made using AutoMontage extended-focus software, using JVC KY-F75U digital camera, Leica Z16 APOA microscope, and 1× objective lens. Images were post-processed with Abobe Photoshop CS3 Extended. The individual wasp images are archived in Specimage (specimage.osu.edu).

Genomic DNA was extracted from ethanol-preserved specimens using the DNeasy Blood & Tissue Kit (Qiagen, Germantown, MD; cat. num. 69506) and following the protocol used by Taekul et al. (2014). A segment of the mitochondrial COI gene region was amplified with PCR using the scelionid-specific primers of Gariepy et al. (2014). PCRs were carried out in 50 μL containing 25 μL GoTaq Green Master Mix, 2× (Promega, USA), 0.5 μL of 100 μM primers and 2.5 μL of genomic DNA. Thermocycling conditions include initial denaturation at 94 °C for 5 min, followed by 35 cycles of 1 min at 94 °C, 1 min at the primers’ annealing temperature, and 1.5 min of elongation at 72 °C, and ending with an additional extension of 72 °C for 3 min. Amplicons were directly sequenced in both directions with forward and reverse primers by Genewiz (South Plainfield, NJ). Chromatograms were assembled with Sequencher v4.0 (Gene Codes Corporation, Ann Arbor, MI). All the amplified sequences are deposited in GenBank (Suppl. material 2). Sequences were aligned in Geneious 11.1.4 using the Geneious alignment algorithm. The aligned sequences were then analyzed using the neighbor-joining algorithm and RAxML as implemented in Geneious 1.1.4 with the corresponding sequence from *Trissolcusbasalis* (Wollaston) (Hymenoptera: Platygastridae, Telenominae) used as an outgroup to root the tree.

## Results

**Figure 1.**
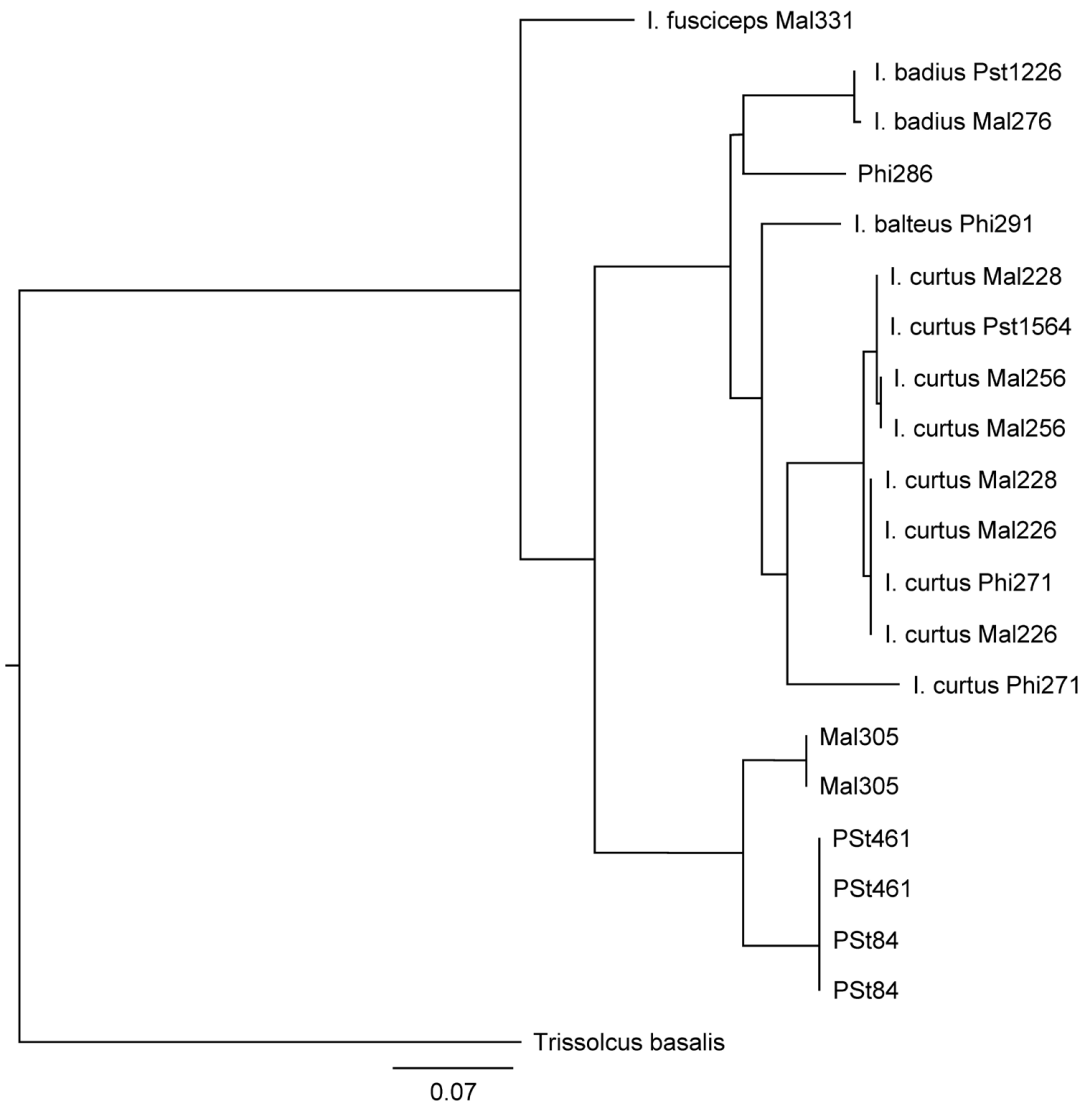
Results of RAxML analysis of COI sequences.

The nucleotide alignment file and GenBank accession numbers are included in Suppl. materials 1, 2 respectively. The results of the RAxML analysis are presented in Figure 1. Both the RAxML and neighbor-joining trees grouped the 20 samples into the same seven groups:

1) Mal305

2) PSt84 + PSt461

3) Mal331 (*Idrisfusciceps* sp. n.)

4) Phi286

5) Mal276 + PSt1226 (*Idrisbadius* sp. n.)

6) Phi 291 (*Idrisbalteus* sp. n.)

7) Mal228 + Mal256 + PSt1564 + Mal226 + Phi271 (*Idriscurtus* sp. n.)

Within each grouping of more than one sequenced sample the average pairwise percentage identity of the sequences was 99.443% (99.142–100%). The average between-group pairwise percentage identity was 88.154% (87.016–90.207%). The average percent identity with the outgroup, *Trissolcusbasalis*, was 79.2%. In one egg sac, Phi271, the two specimens sequenced were identical in only 91.3% of the sequence, suggesting either two different species or a relatively high level of intraspecific variation. Unfortunately, no adults emerged from these eggs so it was impossible to distinguish between the two possibilities on the basis of morphological characters.

Five of the seven groupings of molecular samples were represented by adults, and all but one of these are described below as new species. The one not described could not be distinguished on the basis of morphology alone from *I.badius*. The low level of sequence identity (86.5%) strongly suggests that they are not conspecific, but in lieu of finding any morphological distinction, we decided to refrain from describing it. None of these new species were identifiable on the basis of the key in Lê (2000) nor through examination of images of holotypes in Talamas et al. (2017) and Talamas and Pham (2017).

### Idris badius

Taxon classificationAnimaliaAraneaePholcidae

Johnson & Chen sp. n.

http://zoobank.org/E8EC0A7D-7214-45EE-B0BD-AB8622FF7267

Figures 2 , 6 , 10

#### Description.

Body length: 0.81 mm. Head color: brown. Mesosoma color: brown. Metasoma color: brown.

Head shape in frontal view: ovoid, distinctly wider than high. Head width/mesosomal width: 1.34–1.44. Sculpture of upper frons, vertex: finely coriaceous reticulate. Position of lateral ocellus: contiguous with inner orbit of compound eye. Central keel of frons: present. Length of central keel of frons: extending dorsally half distance to median ocellus. Speculum: present. Striae on lower frons: absent. Setation of compound eyes: eyes distinctly setose.

Size of A3: subequal in length, width to A2. Shape of A3: length greater than width.

Length/width mesoscutum: 0.74–0.83. Sculpture of mesoscutum: finely reticulate, setal bases pustulate. Notauli: absent. Sculpture of mesoscutellum: finely reticulate, setal bases pustulate. Sculpture of metascutellum: smooth. Propodeal armature: lateral propodeal area produced dorsomedially into small tooth.

Wing development: fully developed, macropterous. Fore wing patterning: fore wing hyaline throughout. Marginal fringe of fore wing: present, short. Length of bristles on submarginal vein: short, barely reaching beyond costal margin of wing. Basal vein: well-defined, straight, lightly pigmented. Length of stigmal vein: elongate, extending nearly to middle of fore wing. Length of postmarginal vein: extremely short, subequal in length to marginal vein.

Metasoma length/body length: 0.47–0.48. Sculpture of T1: longitudinally costate. Sculpture of T2: longitudinally costate in medial third, finely reticulate along lateral margin, elsewhere smooth. Length T3/length T2: 2.21. Sculpture of T4–T5: finely reticulate basally, smooth apically. Setation of T3: lateral thirds of tergite moderately setose through, median third nearly glabrous, with sparse apical transverse band of setae.

**Figures 2–5.**
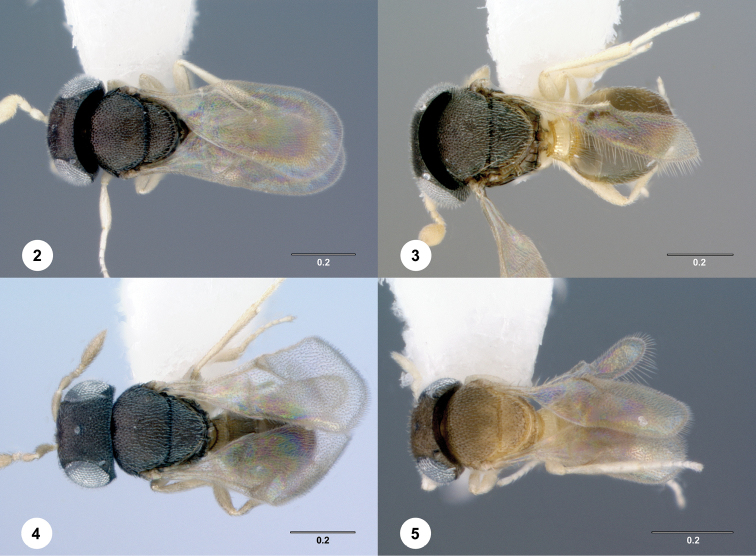
Dorsal habitus. **2***Idrisbadius* sp. n. **3***I.balteus* sp. n. **4***I.curtus* sp. n. **5***I.fusciceps* sp. n. Scale bars in millimeters.

#### Diagnosis.

This species runs to *I.nautalis* Kozlov & Lê in the keys of Kozlov and Lê (1987) and Lê (2000), but differs in the absence of longitudinal striae on T3. It is morphologically indistinguishable from the specimens of sample Mal305 collected at the same time and place, but reared from the eggs of *Tissahamiagombak*. The COI sequences, however, have only 86.5% identity, and this must be used to establish their identity.

#### Host.

Eggs of *Nipisaphyllicola* (Deeleman-Reinhold) (ZFMK, Mal 276) (Fig. 14), *Panjangehamiguitan* Huber (ZFMK, PSt1226 = Ar 13012) (Araneae: Pholcidae).

#### Etymology.

The specific epithet *badius* refers the rich brown color of the body and is intended as an adjective.

#### Material examined.

**Holotype***female*: **MALAYSIA**: Perak, Gunung Liang, forest along river, 250 m a.s.l., 3°47.7'N 101°32.0'E, 22.ii.2015, B. A. Huber, A.R.M. Ghazali & K. A. Braima, ex: egg of *Nipisaphyllicola* (Deeleman-Reinhold), OSUC270822. **Paratypes**: **MALAYSIA**: 3 females, 1 male with same data as holotype, OSUC270823, 420837–420838, 627622. **PHILIPPINES**: Mindanao, Davao Oriental, 580 m a.s.l., 6.6805N, 126.1591E, site 3, Mount Hamiguitan Wildlife Sanctuary (access Governor Generoso), 13.ii.2015, M. A. Responte, ex egg of *Panjangehamiguitan* Huber (1 female, OSUC627625).

### Idris balteus

Taxon classificationAnimaliaAraneaePholcidae

Johnson & Chen sp. n.

http://zoobank.org/04E0C39B-2CAE-4302-8148-413299BE60AE

Figures 3 , 7 , 11

#### Description.

Body length: 0.85–0.99 mm. Head color: dark brown. Mesosoma color: dark brown. Metasoma color: first segment yellow, second segment brownish yellow, otherwise brown.

Head shape in frontal view: ovoid, distinctly wider than high. Head width/mesosomal width: 1.24–1.31. Sculpture of upper frons, vertex: finely coriaceous reticulate. Position of lateral ocellus: contiguous with inner orbit of compound eye. Central keel of frons: present. Length of central keel of frons: extending dorsally half distance to median ocellus. Speculum: present. Striae on lower frons: with short striae flanking speculum. Setation of compound eyes: eyes distinctly setose.

Size of A3: distinctly smaller than A2. Shape of A3: length greater than width.

Length/width mesoscutum: 0.72. Sculpture of mesoscutum: finely reticulate, setal bases pustulate. Notauli: absent. Sculpture of mesoscutellum: finely reticulate, setal bases pustulate. Sculpture of metascutellum: smooth. Propodeal armature: lateral propodeal area produced dorsomedially into small tooth.

Wing development: fully developed, macropterous. Fore wing patterning: fore wing hyaline throughout. Marginal fringe of fore wing: present, short. Length of bristles on submarginal vein: short, barely reaching beyond costal margin of wing. Basal vein: well-defined, straight, lightly pigmented. Length of stigmal vein: elongate, extending nearly to middle of fore wing. Length of postmarginal vein: extremely short, subequal in length to marginal vein.

Metasoma length/body length: 0.45–0.48.Sculpture of T1: longitudinally costate. Sculpture of T2: longitudinally costate in medial third, finely reticulate along lateral margin, elsewhere smooth. Sculpture of T3: finely reticulate, with weak irregularly longitudinal rugulae medially. Length T3/length T2: 1.81–2.17. Sculpture of T4–T5: finely reticulate basally, smooth apically. Setation of T3: lateral thirds of tergite moderately setose through, median third nearly glabrous, with sparse apical transverse band of setae.

#### Diagnosis.

In the keys of Kozlov and Lê (1987) and Lê (2000) this species runs to *I.nautalis*, but differs from that species in the lack of longitudinal striae on T3 and the uniform coloration of the metasoma. Distinguished from many species of *Idris* by the xanthic first segment of the metasoma. The COI sequence will serve to help distinguish this species from others with the same character.

#### Host.

*Panjangecamiguin* Huber (ZFMK, Phi291) (Araneae: Pholcidae) (Fig. 15).

#### Etymology.

The specific epithet *balteus*, a Latin word for belt, refers to the golden base of the metasoma. It is intended as a noun in apposition.

#### Material examined.

**Holotype***female*: **PHILIPPINES**: Bohol, near Loboc, above Loboc River, forest near caves, ~250 m a.s.l., ~9.655N, 124.015E, 5.iii.2014, B. A. Huber, ex egg of *Panjangecamiguin* Huber, OSUC270828. **Paratypes**: **PHILIPPINES**: 3 females, 1 male with same data as holotype, OSUC270829, 420844–420845, 627631). **Other material**: 1 broken female with same data as holotype, OSUC270830.

**Figures 6–9.**
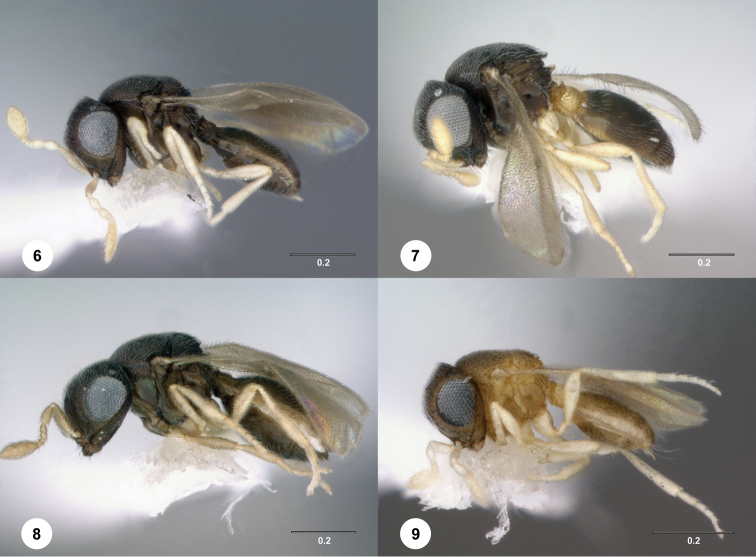
Lateral habitus. **6***Idrisbadius* sp. n. **7***I.balteus* sp. n. **8***I.curtus* sp. n. **9***I.fusciceps* sp. n. Scale bars in millimeters.

### Idris curtus

Taxon classificationAnimaliaAraneaePholcidae

Johnson & Chen sp. n.

http://zoobank.org/C8DA9FB6-A843-4D33-9475-5E3FE090068C

Figures 4 , 8 , 12

#### Description.

Body length: 0.83–0.89 mm. Head color: brown. Mesosoma color: brown. Metasoma color: brown

Head shape in frontal view: ovoid, distinctly wider than high. Head width/mesosomal width: 1.20–1.27. Sculpture of upper frons, vertex: finely coriaceous reticulate. Position of lateral ocellus: contiguous with inner orbit of compound eye. Central keel of frons: present. Length of central keel of frons: extending dorsally half distance to median ocellus. Speculum: present. Striae on lower frons: with short striae flanking speculum. Setation of compound eyes: eyes distinctly setose.

Size of A3: distinctly smaller than A2. Shape of A3: length greater than width.

Length/width mesoscutum: 0.65–0.79. Sculpture of mesoscutum: finely reticulate, setal bases pustulate. Notauli: absent. Sculpture of mesoscutellum: finely reticulate, setal bases pustulate. Sculpture of metascutellum: smooth. Propodeal armature: lateral propodeal area produced dorsomedially into small tooth.

Wing development: fully developed, macropterous. Fore wing patterning: fore wing hyaline throughout. Marginal fringe of fore wing: present, short. Length of bristles on submarginal vein: short, barely reaching beyond costal margin of wing. Basal vein: well-defined, straight, lightly pigmented. Length of stigmal vein: elongate, extending nearly to middle of fore wing. Length of postmarginal vein: extremely short, subequal in length to marginal vein.

Metasoma length/body length: 0.52–0.57. Sculpture of T1: longitudinally costate. Sculpture of T2: longitudinally costate in medial third, finely reticulate along lateral margin, elsewhere smooth. Length T3/length T2: 1.87–2.33. Sculpture of T4–T5: finely reticulate basally, smooth apically. Setation of T3: lateral thirds of tergite moderately setose through, median third nearly glabrous, with sparse apical transverse band of setae.

**Figures 10–13.**
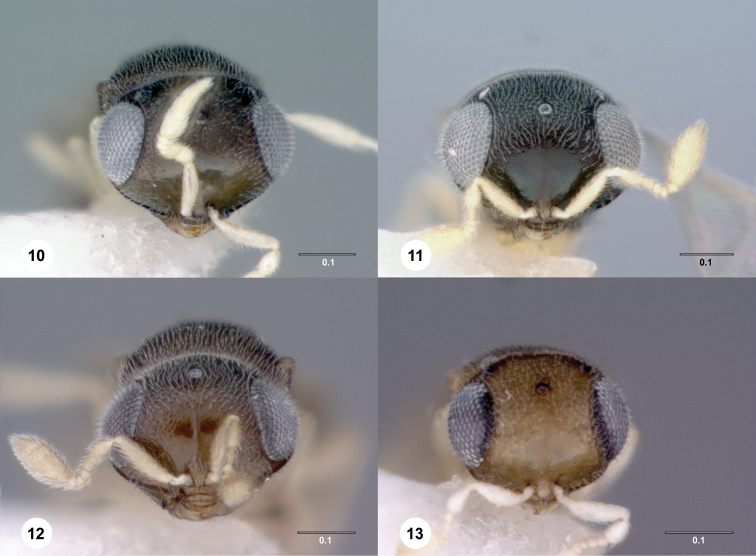
Head, anterior view. **10***Idrisbadius* sp. n. **11***I.balteus* sp. n. **12***I.curtus* sp. n. **13***I.fusciceps* sp. n. Scale bars in millimeters.

#### Diagnosis.

Very similar to *I.badius*, distinguished by the distinctly shorter third antennomere (compared with A2). As with *I.badius*, this species runs to *I.nautalis* Kozlov & Lê in the keys of Kozlov and Lê (1987) and Lê (2000). It differs in the absence of longitudinal striae on T3. Distinguished from other species of *Idris* by its COI sequence.

#### Host.

*Calapnitanunezae* Huber (ZFMK, Phi271), *Panjangecamiguin* Huber (ZFMK, PSt1564 = Ar 15064), *Tissahamiabukittimah* (Huber) (ZFMK, Mal 256) (Fig. 16), *Uthinaluzonica* Simon (ZFMK, Mal228 = Ar 19637 and Mal226) (Araneae: Pholcidae).

#### Etymology.

The name *curtus*, Latin for short, refers to relative length of the first flagellomere of the female antenna. It is intended to be used as an adjective.

#### Material examined.

**Holotype***female*: **SINGAPORE**: 50 m a.s.l., 1°21.6'N 103°46.7'E, Dairy Farm Nature Park, 15.ii.2015, B. A. Huber & J. Koh, reared ex egg of *Tissahamiabukittimah* (Huber), OSUC270831. **Paratypes**: **SINGAPORE**: 6 females, 2 males, with same data as holotype, OSUC270832, 270833, 420829–420831, 420833, 627623, 627624. 1 female, 2 males with same data as holotype except reared ex egg of *Uthinaluzonica* Simon, OSUC420836, 6217633, 627634. **PHILIPPINES**: Visayas, Bohol, Bilar, Barangay Riverside, 440 m a.s.l., 9.7052N, 124.1253E, 15.vi.2015, M.R.B. Dacar; reared ex egg of *Panjangecamiguin* Huber, 1 female, 1 male, OSUC420834, 627632. **Other material**: **SINGAPORE**: 50 m a.s.l., 1°21.6'N 103°46.7'E, Dairy Farm Nature Park, 15.ii.2015, reared, B. A. Huber & J. Koh, ex egg of *Tissahamiabukittimah* (Huber), female, OSUC420832 (broken specimen); ex egg of *Uthinaluzonica* Simon, male, OSUC420835 (broken). The remaining material is based on DNA sequences from pupae. **SINGAPORE**: Upper Selatar Reservoir Park (1°21.3'N, 103°48.4'E), 20 m a.s.l., 15.ii.2015 (B.A. Huber, D. Court). Host: *Uthinaluzonica* Simon. **PHILIPPINES**: Mindanao, Mt. Matutum, Kawit Forest, ‘site 1’, (6.338N, 125.104E), 950 m a.s.l., along brook, on leaves, 13.ii.2014, B. A. Huber, ex egg of *Calapnitanunezae* Huber.

### Idris fusciceps

Taxon classificationAnimaliaAraneaePholcidae

Johnson & Chen sp. n.

http://zoobank.org/F316941C-D414-4DC1-9420-129ADBC8192E

Figures 5 , 9 , 13

#### Description.

Body length: 0.58–0.66 mm.

Head color: light brown. Mesosoma color: brownish yellow, contrasting with darker color of head. Metasoma color: brownish yellow.

Head shape in frontal view: ovoid, distinctly wider than high. Head width/mesosomal width: 1.17–1.24. Sculpture of upper frons, vertex: pustulate. Position of lateral ocellus: separated from inner orbit of compound eye by approximately 1 ocellar diameter. Central keel of frons: present. Length of central keel of frons: extending dorsally one-third distance to median ocellus. Speculum: present. Striae on lower frons: absent. Setation of compound eyes: eyes distinctly setose.

Size of A3: distinctly smaller than A2. Shape of A3: width greater than length.

Length/width mesoscutum: 0.64–0.72. Sculpture of mesoscutum: finely reticulate, setal bases pustulate. Notauli: present, short. Sculpture of mesoscutellum: finely reticulate, setal bases pustulate. Sculpture of metascutellum: rugulose. Propodeal armature: lateral propodeal area produced dorsomedially into small tooth.

Wing development: fully developed, macropterous. Fore wing patterning: fore wing hyaline throughout. Marginal fringe of fore wing: present, short. Length of bristles on submarginal vein: elongate, extending beyond costal margin by distance more than half their length. Basal vein: well-defined, straight, lightly pigmented. Length of stigmal vein: elongate, extending nearly to middle of fore wing. Length of postmarginal vein: extremely short, subequal in length to marginal vein.

Metasoma length/body length: 0.44–53. Sculpture of T1: longitudinally costate. Sculpture of T2: longitudinally costate in medial third, finely reticulate along lateral margin, elsewhere smooth. Sculpture of T3: finely reticulate, with weak irregularly longitudinal rugulae medially. Length T3/length T2: 1.56–1.60. Sculpture of T4–T5: finely reticulate basally, smooth apically. Setation of T3: lateral thirds of tergite moderately setose through, median third nearly glabrous, with sparse apical transverse band of setae.

#### Diagnosis.

In the keys of Kozlov and Lê (1987) and Lê (2000), this species comes closest to *I.oobius* Kozlov & Lê, particularly in the weak sculpture of T3 and the uniform coloration of the wings. This species is distinguished from *I.oobius* by the extremely short A3 in comparison to the length of A2. It may be distinguished from the other reared species described herein by the presence of notauli and the dark-colored head; among other morphologically similar species of *Idris* it may be recognized by its COI sequence.

#### Host.

*Belisanakhaosok* Huber (ZFMK, Mal331= Ar 19649) (Araneae: Pholcidae) (Fig. 20).

#### Etymology.

The specific epithet refers to the darker color of the head in comparison with the mesosoma and metasoma. It is to be treated as a noun in apposition.

#### Material examined.

**Holotype**, female: **THAILAND**: Krabi, ~9 km N Krabi town, degraded forest between plantation and rocks, 75 m a.s.l., 8°09.9'N 98°51.7'E, 7.III.2015, B. A. Huber & B. Petcharad, ex egg of *Belisanakhaosok* Huber. OSUC270824. **Paratypes**: **THAILAND**: 8 females, 1 male with same data as holotype (OSUC270825–270827, 420839–420843, 627626).

**Figures 14–20.**
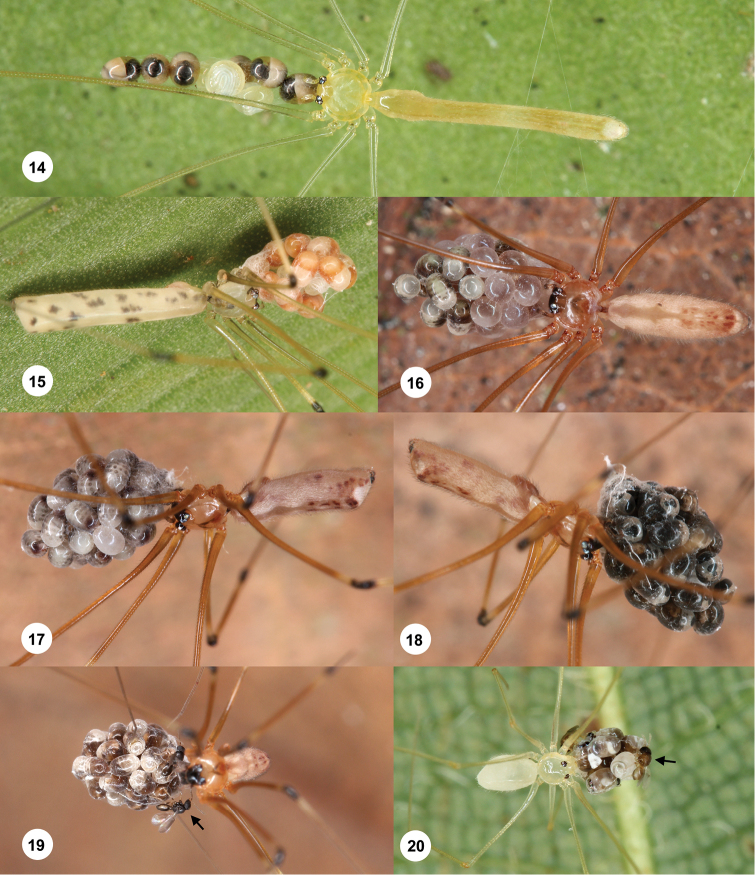
Host spiders with parasitized egg-sacs. **14***Nipisaphyllicola* (Mal276); note that two eggs are not parasitized **15***Panjangecamiguin* (Phi291) **16***Tissahamiabukittimah* (Mal256); note that only some of the eggs are parasitized **17–19***Tissahamiagombak* (Mal305) at different stages of wasp development (6 days lie between **17** and **18** 1 day between **18** and **19**); arrow points at eclosed wasp **20***Belisanakhaosok* (Mal331); arrow points at eclosed wasp.

## Supplementary Material

XML Treatment for Idris badius

XML Treatment for Idris balteus

XML Treatment for Idris curtus

XML Treatment for Idris fusciceps

## Supplementary materials

Supplementary material 1 Fasta file: pholcid Idris Data type: Fasta file This is an open access article distributed under the terms of the Creative Commons Attribution License (CC BY 4.0), which permits unrestricted use, distribution, and reproduction in any medium, provided the original author and source are credited.Norman F. Johnson, Huayan Chen, Bernhard A. Huber

Supplementary material 2 GenBank accessions: pholcid Idris Data type: Microsoft Excel Worksheet (.xlsx) Explanation note: GenBank Accession numbers for DNA sequences.. This is an open access article distributed under the terms of the Creative Commons Attribution License (CC BY 4.0), which permits unrestricted use, distribution, and reproduction in any medium, provided the original author and source are credited.Norman F. Johnson, Huayan Chen, Bernhard A. Huber
