# Supplementary material for: New species of Idris Förster (Hymenoptera, Platygastroidea) from southeast Asia, parasitoids of the eggs of pholcid spiders (Araneae, Pholcidae)
Source: Zookeys. 2018 Dec 31;(811):65–80. doi: 10.3897/zookeys.811.29725 (PMC6323100; doi:10.3897/zookeys.811.29725)
Supplement: Supplementary material 1 [file zookeys-811-065-s001.docx]

Supplementary File 01

>Mal276 - Idris badius

----------GTGACCAAAAAATCAAAATAAATGCTGGTATAAAATGGGGTCTCCCCCTCCTGTTGGGTTAAAGAAAGAA

GTATTTAAATTTCGGTCAGTTAATAATATTGTTAATCCCCCTGCTAATACGGGTAATGATAATAATAAAAGAATTGTTGT

GATAAAAACAGATCAACAAAATAATGAAATGTTTTTTAT--ATAAATTTTATTTGATTTTATATTTAAAATTGTACAAAG

AAAATTAATTGATCTAAGGATTGAGGAAATTCCTGCAATATGAAGGGAGAAGATTGTAAAATCAATTGAAGGATTATTTT

GTATAGATAAGGGGGGGTAAATGGTTCATCCTGTTCCTGTTCCTCTCCCACAGATATTTCTATAGATTAGTAAGATAATA

GAAGGTGGCAATAATCAAAATCTTATATTATTTAAACGTGGAAAGGCTATATCTGGAGATGCTAGTATTAGTGGGACTAA

TCAATTTCCAAACCCCCCTAATATTAAAGGCATAACTATAAAGAAAATTATAATAAAGGCGTGAGAAGTTACAATAGAAT

TATAAA-TTTGATCATTTCCAATTAATATTCCAGGAACTCTTAATTC---------------

>Mal256-1 - Idris curtus

----------GTGACCAAAAAATCAGAATAGATGTTGGTATAAAATAGGATCCCCTCCTCCCGTTGGGTTAAAGAAGGAA

GTATTTAAATTTCGATCAGTTAATAATATTGTTAGTCCTCCTGCTAATACAGGTAGGGATAGTAATAAAAGAATTGTTGT

AATAAAAATAGATCAACAAAATAATGAAATATTTTTTAT--ATAAATTTTATTAGACTTTATATTTAAAATTGTGCAAAG

AAAATTAATGGATCTAAGAATTGAAGAGATTCCTGCAATATGAAGAGAGAAAATTGTAAAATCAATTGAAGGGTTAGTTT

GTATTGATAGGGGGGGATAAACAGTTCATCCTGTTCCTGTTCCTCTTCCACAAATATTTCTATAAATTAATAAGATAATA

GAAGGAGGTAATAATCAAAATCTTATGTTATTTAAGCGTGGAAATGCTATATCTGGGGAGGCCACTATTAATGGGACTAG

TCAATTTCCAAACCCTCCTAACATTAGGGGTATAACTATAAAGAAAATTATAATAAAAGCGTGTGAAGTTACAATTGAAT

TATAAA-TTTGATCATTTCCAATTAATATTCCGGGAACTCTTAATTC---------------

>Mal256-2 - Idris curtus

----------GTGACCAAAAAATCAGAATAGATGTTGGTATAAAATAGGATCCCCTCCTCCCGTTGGGTTAAAGAAGGAA

GTATTTAAATTTCGATCAGTTAATAATATTGTTAGTCCTCCTGCTAATACAGGTAGGGATAGTAATAAAAGAATTGTTGT

AATAAAAATAGATCAACAAAATAATGAAATATTTTTTAT--ATAAATTTTATTAGACTTTATATTTAAAATTGTGCAAAG

AAAATTAATGGATCTAAGAATTGAAGAGATTCCTGCAATATGAAGAGAGAAAATTGTAAAATCAATTGAAGGGTTAGTTT

GTATTGATAGGGGGGGATAAACAGTTCATCCTGTTCCTGTTCCTCTTCCACAAATATTTCTATAAATTAATAAGATAATA

GAAGGAGGTAATAATCAAAATCTTATGTTATTTAAGCGTGGAAATGCTATATCTGGGGAGGCCACTATTAATGGGACTAG

TCAATTTCCAAACCCTCCTAACATTAGGGGTATAACTATAAAGAAAATTATAATAAAAGCGTGTGAAGTTACAATTGAAT

TATAAA-TTTGATCATTTCCAATTAATATTCCGGGAACTCTTAATTCTATTCG---------

>PSt1226 - Idris badius

----------GTGACCAAAAAATCAAAATAAATGCTGGTATAAAATGGGGTCTCCCCCTCCTGTTGGATTAAAGAAAGAA

GTATTTAAATTTCGGTCAGTTAATAATATTGTTAATCCCCCTGCTAATACGGGTAATGATAATAATAAAAGAATTGTTGT

GATAAAAACAGATCAACAAAATAATGAAATGTTTTTTAT--ATAAATTTTATTTGATTTTATATTTAAAATTGTACAAAG

AAAATTAATTGATCTAAGGATTGAGGAAATTCCTGCAATATGAAGGGAGAAGATTGTAAAATCAATTGAAGGATTATTTT

GTATAGATAAGGGGGGGTAAATGGTTCATCCTGTTCCTGTTCCTCTCCCACAGATATTTCTATAGATTAGTAAGATAATA

GAAGGTGGTAATAATCAAAATCTTATATTATTTAAACGTGGAAAGGCTATATCTGGAGATGCTAGTATTAGTGGGACTAA

TCAATTTCCAAACCCCCCTAATATTAAAGGCATAACTATAAAGAAAATTATAATAAAGGCGTGAGAAGTTACAATAGAAT

TATAAA-TTTGATCATTTCCAATTAATATTCCAGGAACTCTTAATTC---------------

>Mal331 Idris fusciceps

-------------ACCAAAAAATCAGAATAGGTGCTGGTATAAAATTGGATCCCCTCCTCCATTAGGGTTAAAAAAGGAT

GTATTTAAATTTCGATCTGTTAGTAATATTGTTAGTCCTCCAGCTAGAACAGGAAGGGATAATAATAGTAGAATTGTTGT

GATGAAAATTGATCAGCAAAATAATGAAATATTTTTTAT--AAAAATTTTGTTTGATTTTATATTTAGAATTGTACATAG

AAAGTTAATTGATCTAAGAATTGAAGAAATTCCTGCAATATGTAATGAAAAAATTGTAAAATCAATTGATGGGTTAGTTT

GTATTGATAAAGGAGGGTAAACAGTTCATCCAGTTCCTGTTCCTCTTCCGCAAATATTTCTATAGATTAATAAAATAATA

GAAGGAGGTAAAAGTCAAAATCTTATATTATTTAAACGTGGGAATGCTATATCTGGAGAGGATAATATTAGGGGGATTAA

TCAATTTCCAAATCCTCCTAATATTAAAGGTATAACTATAAAAAAAATCATAATAAAAGCATGGGATGTAACAATTGAAT

TATAAA-TTTGGTCATTTCCAATTAATATTCCTGGTACTCTTAA------------------

>Mal305-2 - Idris sp.

-------------ACCAAAAAATCAAAATAAATGCTGATATAAAATTGGATCTCCACCTCCTGTTGGGTTAAAAAATCTT

GTGTTTAGATTTCGGTCTGTTAACAATATTGTTAGTCCCCCTGCTAAAACAGGTAAAGATAATAATAATAAAATTGTTGT

AATGAAAATAGATCAACAAAATAATGAAATATTTTTTAT--AATAATTTTATTAGATTTTATATTTAAAATTGTACATAA

AAAATTAATTGATCTTAAAATTGATGAAATACCTGCAATATGAAGAGAAAAAATAGTAAAATCAATTGATGGGTTATTTT

GTATTGATAAAGGGGGATAAACTGTTCATCCAGTTCCAGTACCTCTTCCACAGATATTTCTATAAATTAATAATAAGATT

GAGGGGGGTAATAGTCAAAATCTTATATTATTTAAACGTGGGAAAGCTATATCTGGTGAAGATAATATTAATGGAACTAA

TCAATTTCCAAATCCTCCTAATATTAAAGGTATTACTATAAAAAAAATTATAATAAAAGCGTGGGAAGTAACAATTGAAT

TATAAA-TTTGGTCATTACCAATTAGTATTCCTGGTACTCTTAATTC---------------

>Mal305-1 - Idris sp.

----------GTGACCAAAAAATCAAAATAAATGCTGATATAAAATTGGATCTCCACCTCCTGTTGGGTTAAAAAATCTT

GTGTTTAGATTTCGGTCTGTTAACAATATTGTTAGTCCCCCTGCTAAAACAGGTAAAGATAATAATAATAAAATTGTTGT

AATGAAAATAGATCAACAAAATAATGAAATATTTTTTAT--AATAATTTTATTAGATTTTATATTTAAAATTGTACATAA

AAAATTAATTGATCTTAAAATTGATGAAATACCTGCAATATGAAGAGAAAAAATAGTAAAATCAATTGATGGGTTATTTT

GTATTGATAAAGGGGGATAAACTGTTCATCCAGTTCCAGTACCTCTTCCACAGATATTTCTATAAATTAATAATAAGATT

GAGGGGGGTAATAGTCAAAATCTTATATTATTTAAACGTGGGAAAGCTATATCTGGTGAAGATAATATTAATGGAACTAA

TCAATTTCCAAATCCTCCTAATATTAAAGGTATTACTATAAAAAAAATTATAATAAAAGCGTGGGAAGTAACAATTGAAT

TATAAA-TTTGGTCATTACCAATTAGTATTCCTGGTACTCTTAATTC---------------

>Phi291 - Idris sp.

-------------ACCAAAAAATCAAAATAAATGTTGGTATAAAATAGGATCTCCTCCTCCTGTTGGATTAAAGAAAGAA

GTATTTAGATTTCGATCGGTTAATAATATTGTTAATCCTCCTGCTAATACAGGTAGTGATAATAATAAAAGAATTGTTGT

AATAAAAATAGATCAACAAAATAATGAGATGTTTTTTAT--ATAAACTTTATTAGATTTTATATTTAAAATTGTACAAAG

AAAATTAATTGATCTAAGGATTGAAGAAATTCCTGCAATATGAAGAGAAAAAATTGTAAAATCAATTGATGGATTAGTTT

GTATAGACAAAGGTGGATAAACAGTTCATCCTGTTCCTGTTCCTCTTCCACAAATATTTCTATAAATTAATAAAATAATA

GAAGGGGGTAATAGTCAAAATCTTATATTATTTAGTCGTGGAAAAGCTATATCTGGGGAGGCCATTATTAATGGGACTAG

TCAATTTCCAAACCCACCTAATATTAAGGGTATCACTATAAAAAAAATTATGATAAATGCGTGAGAAGTTACAATAGAAT

TATAAA-TTTGATCATTTCCAATTAATATTCCTGGAACTCTTAATTC---------------

>PSt1564 - Idris curtus

----------GTGACCAAAAAATCAGAATAGATGTTGGTATAAAATAGGATCCCCTCCTCCCGTTGGGTTAAAGAAGGAA

GTATTTAAATTTCGATCAGTTAATAATATTGTTAGTCCTCCTGCTAATACAGGTAGGGATAGTAATAAAAGAATTGTTGT

AATAAAAATAGATCAACAAAATAATGAAATATTTTTTAT--ATAAATTTTATTAGATTTTATATTTAAAATTGTGCAAAG

AAAATTAATGGATCTAAGAATTGAAGAGATTCCTGCAATATGAAGAGAGAAAATTGTAAAATCAATTGAAGGGTTAGTTT

GTATAGATAGGGGGGGATAAACAGTTCATCCTGTTCCTGTTCCTCTTCCACAAATATTTCTATAAATTAATAAGATAATA

GAAGGAGGTAATAATCAAAATCTTATGTTATTTAAGCGTGGAAATGCTATATCTGGGGAGGCCACTATTAATGGGACTAG

TCAATTTCCAAACCCTCCTAACATTAGGGGTATAACTATAAAGAAAATTATAATAAAAGCGTGTGAAGTTACAATTGAAT

TATAAA-TTTGATCATTTCCAATTAATATTCCGGGAACTCTTAATTCTAT------------

>Mal228-1 - Idris curtus

-------------ACCAAAAAATCAGAATAGATGTTGGTATAAAATAGGATCCCCTCCTCCCGTTGGGTTAAAGAAGGAA

GTATTTAAATTTCGATCAGTTAATAATATTGTTAGTCCTCCTGCTAATACAGGTAGGGATAGTAATAAAAGAATTGTTGT

AATAAAAATAGATCAACAAAATAATGAAATATTTTTTAT--ATAAATTTTATTAGATTTTATATTTAAAATTGTGCAAAG

AAAATTAATGGATCTAAGAATTGAAGAGATTCCTGCAATATGAAGAGAGAAAATTGTAAAATCAATTGAAGGGTTAGTTT

GTATAGATAGGGGGGGATAAACAGTTCATCCTGTTCCTGTTCCTCTTCCACAAATATTTCTATAAATTAATAAGATAATA

GAAGGAGGTAATAATCAAAATCTTATGTTATTTAAGCGTGGAAATGCTATATCTGGGGAGGCCACTATTAATGGGACTAG

TCAATTTCCAAACCCTCCTAACATTAGGGGTATAACTATAAAGAAAATTATAATAAAAGCGTGTGAAGTTACAATTGAAT

TATAAA-TTTGATCATTTCCAATTAATATTCCGGGAACTCTTAATTCTAT------------

>Mal228-2 - Idris curtus

----------GTGACCAAAAAATCAGAATAGATGTTGGTATAAAATAGGATCCCCCCCTCCTGTTGGGTTAAAGAAGGAA

GTATTTAAATTTCGATCAGTTAATAATATTGTTAGTCCTCCTGCTAATACAGGTAGGGATAGTAATAAAAGAATTGTTGT

AATAAAAATAGATCAACAAAATAATGAAATATTTTTTAT--ATAAATTTTATTAGATTTTATATTTAAAATTGTGCAAAG

AAAATTAATGGATCTAAGAATTGAAGAGATTCCTGCAATATGAAGAGAGAAAATTGTAAAATCAATTGAAGGGTTAGTTT

GTATAGATAGGGGGGGATAAACAGTTCATCCCGTTCCTGTCCCTCTTCCACAAATATTTCTATAAATTAATAGGATAATA

GAAGGAGGTAATAACCAAAATCTTATGTTATTTAAGCGTGGAAATGCTATATCTGGGGAGGCCATTATTAATGGGACTAG

TCAATTTCCAAACCCTCCTAACATTAGGGGTATAACTATAAAGAAAATTATAATAAAAGCGTGTGAAGTTACAATTGAAT

TATAAA-TTTGATCATTTCCAATTAATATTCCGGGAACTCTTAATTCTAT------------

>PSt461-1 - Idris sp.

----------------AAAAAATCAAAATAAATGTTGATATAAAATTGGGTCTCCCCCACCAGTTGGATTAAAAAATCTT

GTGTTTAAGTTTCGGTCTGTTAATAATATTGTTAAGCCTCCTGCTAAAACGGGTAGAGATAATAGTAATAAAATTGTTGT

AATAAAAATAGATCAACAAAATAATGAAATATTTTTTAT--AAAAATTTTGTTAGATTTTATATTTAAAATTGTGCATAA

AAAATTAATTGATCTTAAAATTGATGAAATCCCTGCAATATGAAGAGAAAAAATAGTAAAATCAATTGAGGGATTATTTT

GTATTGATAAAGGGGGATAAACTGTTCATCCAGTTCCTGTACCTCTACCACAAATATTTCTATAAATTAATAATAAGATT

GAGGGGGGTAAAAGTCAAAATCTTATATTATTTAAACGTGGGAAGGCTATGTCTGGGGAAGATAATATTAATGGTACTAA

TCAATTTCCAAACCCTCCTAATATTAAAGGTATTACTATAAAAAAAATTATAATAAAAGCATGGGAAGTAACGATTGAAT

TATAAA-TTTGGTCATTACCAATTAATATTCCTGGGACTCTTAATTC---------------

>PSt461-2 - Idris sp.

----------GTGACCAAAAAATCAAAATAAATGTTGATATAAAATTGGGTCTCCCCCACCAGTTGGATTAAAAAATCTT

GTGTTTAAGTTTCGGTCTGTTAATAATATTGTTAAGCCTCCTGCTAAAACGGGTAGAGATAATAGTAATAAAATTGTTGT

AATAAAAATAGATCAACAAAATAATGAAATATTTTTTAT--AAAAATTTTGTTAGATTTTATATTTAAAATTGTGCATAA

AAAATTAATTGATCTTAAAATTGATGAAATCCCTGCAATATGAAGAGAAAAAATAGTAAAATCAATTGAGGGATTATTTT

GTATTGATAAAGGGGGATAAACTGTTCATCCAGTTCCTGTACCTCTACCACAAATATTTCTATAAATTAATAATAAGATT

GAGGGGGGTAAAAGTCAAAATCTTATATTATTTAAACGTGGGAAGGCTATGTCTGGGGAAGATAATATTAATGGTACTAA

TCAATTTCCAAACCCTCCTAATATTAAAGGTATTACTATAAAAAAAATTATAATAAAAGCATGGGAAGTAACGATTGAAT

TATAAATTTTGGTCATTACCAA----------------------------------------

>PSt84-1 - Idris sp.

----------------AAAAAATCAAAATAAATGTTGATATAAAATTGGGTCTCCCCCACCAGTTGGATTAAAAAATCTT

GTGTTTAAGTTTCGGTCTGTTAATAATATTGTTAAGCCTCCTGCTAAAACGGGTAGAGATAATAGTAATAAAATTGTTGT

AATAAAAATAGATCAACAAAATAATGAAATATTTTTTAT--AAAAATTTTGTTAGATTTTATATTTAAAATTGTGCATAA

AAAATTAATTGATCTTAAAATTGATGAAATCCCTGCAATATGAAGAGAAAAAATAGTAAAATCAATTGAGGGATTATTTT

GTATTGATAAAGGGGGATAAACTGTTCATCCAGTTCCTGTACCTCTACCACAAATATTTCTATAAATTAATAATAAGATT

GAGGGGGGTAAAAGTCAAAATCTTATATTATTTAAACGTGGRAAGGCTATGTCTGGGGAAGATAATATTAATGGTACTAA

TCAATTTCCAAACCCTCCTAATATTAAAGGTATTACTATAAAAAAAATTATAATAAAAGCATGGGAAGTAACGATTGAAT

TATAAA-TTTGGTCATTACCAATTAATATTCCTGGGACTCTTAATTCTAT------------

>PSt84-2 Idris sp.

-------------ACCAAAAAATCAAAATAAATGTTGATATAAAATTGGGTCTCCCCCACCAGTTGGATTAAAAAATCTT

GTGTTTAAGTTTCGGTCTGTTAATAATATTGTTAAGCCTCCTGCTAAAACGGGTAGAGATAATAGTAATAAAATTGTTGT

AATAAAAATAGATCAACAAAATAATGAAATATTTTTTAT--AAAAATTTTGTTAGATTTTATATTTAAAATTGTGCATAA

AAAATTAATTGATCTTAAAATTGATGAAATCCCTGCAATATGAAGAGAAAAAATAGTAAAATCAATTGAGGGATTATTTT

GTATTGATAAAGGGGGATAAACTGTTCATCCAGTTCCTGTACCTCTACCACAAATATTTCTATAAATTAATAATAAGATT

GAGGGGGGTAAAAGTCAAAATCTTATATTATTTAAACGTGGGAAGGCTATGTCTGGGGAAGATAATATTAATGGTACTAA

TCAATTTCCAAACCCTCCTAATATTAAAGGTATTACTATAAAAAAAATTATAATAAAAGCATGGGAAGTAACGATTGAAT

TATAAA-TTTGGTCATTACCAATTAATATTCCTGGGACTCTTAATTCTAT------------

>Mal226-1 - Idris curtus

AAACTTCAGGGTGACCAAAAAATCAGAATAGATGTTGGTATAAAATAGGATCCCCCCCTCCTGTTGGGTT-AAGAAGGAA

GTATTTAAATTTCGATCAGTTAATAATATTGTTAGTCCTCCTGCTAATACAGGTAGGGATAGTAATAAAAGAATTGTTGT

AATAAAAATAGATCAACAAAATAATGAAATATTTTTTAT--ATAAATTTTATTAGATTTTATATTTAAAATTGTGCAAAG

AAAATTAATGGATCTAAGAATTGAAGAGATTCCTGCAATATGAAGAGAGAAAATTGTAAAATCAATTGAAGGGTTAGTTT

GTATAGATAGGGGGGGATAAACAGTTCATCCCGTTCCTGTCCCTCTTCCACAAATATTTCTATAAATTAATAGGATAATA

GAAGGAGGTAATAACCAAAATCTTATGTTATTTAAGCGTGGAAATGCTATATCTGGGGAGGCCATTATTAATGGGACTAG

TCAATTTCCAAACCCTCCTAACATTAGGGGTATAACTATAAAGAAAATTATAATAAAAGCGTGTGAAGTTACAATTGAAT

TATAAA-TTTGATCATTTCCAATTAATATTCCGGGAACTCTTAATTCTATTCGAATTATTGC

>Mal226-2 - Idris curtus

----------GTGACCAAAAAATCAGAATAGATGTTGGTATAAAATAGGATCCCCCCCTCCTGTTGGGTTAAAGAAGGAA

GTATTTAAATTTCGATCAGTTAATAATATTGTTAGTCCTCCTGCTAATACAGGTAGGGATAGTAATAAAAGAATTGTTGT

AATAAAAATAGATCAACAAAATAATGAAATATTTTTTAT--ATAAATTTTATTAGATTTTATATTTAAAATTGTGCAAAG

AAAATTAATGGATCTAAGAATTGAAGAGATTCCTGCAATATGAAGAGAGAAAATTGTAAAATCAATTGAAGGGTTAGTTT

GTATAGATAGGGGGGGATAAACAGTTCATCCCGTTCCTGTCCCTCTTCCACAAATATTTCTATAAATTAATAGGATAATA

GAAGGAGGTAATAACCAAAATCTTATGTTATTTAAGCGTGGAAATGCTATATCTGGGGAGGCCATTATTAATGGGACTAG

TCAATTTCCAAACCCTCCTAACATTAGGGGTATAACTATAAAGAAAATTATAATAAAAGCGTGTGAAGTTACAATTGAAT

TATAAA-TTTGATCATTTCCAATTAATATTCCGGGAACTCTTAATTCTAT------------

>Phi271-1 - Idris curtus

----------GTGACCAAAAAATCAGAATAGATGTTGGTATAAAATAGGATCCCCCCCTCCTGTTGGGTTAAAGAAGGAA

GTATTTAAATTTCGATCAGTTAATAATATTGTTAGTCCTCCTGCTAATACAGGTAGGGATAGTAATAAAAGAATTGTTGT

AATAAAAATAGATCAACAAAATAATGAAATATTTTTTAT--ATAAATTTTATTAGATTTTATATTTAAAATTGTGCAAAG

AAAATTAATGGATCTAAGAATTGAAGAGATTCCTGCAATATGAAGAGAGAAAATTGTAAAATCAATTGAAGGGTTAGTTT

GTATAGATAGGGGGGGATAAACAGTTCATCCCGTTCCTGTCCCTCTTCCACAAATATTTCTATAAATTAATAGGATAATA

GAAGGAGGTAATAACCAAAATCTTATGTTATTTAAGCGTGGAAATGCTATATCTGGGGAGGCCATTATTAATGGGACTAG

TCAATTTCCAAACCCTCCTAACATTAGGGGTATAACTATAAAGAAAATTATAATAAAAGCGTGTGAAGTTACAATTGAAT

TATAAA-TTTGATCATTTCCAATTAATATTCCGGGAACTCTTAATTCTAT------------

>Phi271-2 - Idris curtus

-------AGGGTGACCAAAAAATCAGAATAAGTGTTGGTANAGAATAGGGTCCCCCCCACCGGTTGGGTTAAAAAATGAA

GTGTTTAGGTTTCGGTCAGTTAATAATATTGTTAGTCCTCCTGCTAATACAGGTAATGATAATAGTAGTAAAATTGTTGT

AATAAAAATAGATCAACAAAATAATGAGATGTTTTTTAT--ATAAACTTTGTTTGATTTTATATTTAGGATTGTACAAAG

AAAATTAATTGATCTAAGGATTGAAGAGATTCCTGCAATATGGAGGGAGAAAATTGTAAAATCAATTGATGGGTTAGTTT

GTATAGATAAGGGGGGATAAACAGTTCATCCTGTTCCCGTCCCTCTTCCACAAATATTTCTATAAATTAATAAAATAATA

GAAGGAGGTAATAACCAAAATCTTATGTTATTTAAGCGTGGAAAAGCTATGTCTGGGGAAGCTATTATTAATGGGACTAG

TCAATTTCCGAACCCTCCTAATATAAGAGGTATAACTATAAAAAAAATTATAATAAAAGCGTGGGAAGTTACAATAGAAT

TATAAA-TTTGATCATTTCCAATTAACATTCCTGGGACTCTTAATTCTAT------------

>Phi286 - Idris balteus

----------GTGACCAAAAAATCAAAATAAATGTTGATATAAAATAGGATCCCCCCCTCCTGTTGGATTAAAAAATGAA

GTATTTAAATTTCGGTCAGTTAATAATATTGTTAATCCTCCTGCTAATACGGGTAATGATAATAATAAAAGAATTGTTGT

AATAAAGATAGATCAACAGAATAATGAGATATTTTTTAT--ATAAATTTTGTTAGATTTTATATTTAAAATTGTACAAAG

AAAGTTAATTGATCTAAGGATTGAAGAAATCCCTGCAATATGAAGAGAAAAAATTGTAAAATCAATTGAAGGATTAGTTT

GTATAGATAAAGGGGGGTATACCGTTCATCCTGTTCCCGTTCCTCTTCCACAGATATTACTATAAATTAATAAAATAATA

GATGGGGGTAATAATCAAAATCTTATATTATTTAAGCGTGGAAAGGCTATATCTGGAGAAGCTAATATTAATGGCACTAA

TCAATTTCCAAATCCCCCTAATATTAAAGGTATAACTATAAAAAAAATTATAATAAAAGCATGAGAGGTTACAATAGAGT

TATAAA-TTTGATCATTTCCAATTAGTATACCTGGAACTCTTAATTCTAT------------

>Trissolcus_basalis

----------GTGTCCAAAAAATCAAAAAAGATGTTGGTAGAGAACTGGGTCTCCTCCTCCTCCAGGATTAAAAAATGAT

GTATTTAAATTTCGATCTGATAAAATTATTGTAATAGCTCCAGCTAAAACTGGTAATGATAATAATAAAAGAATTGTAGT

AATTAATACTGATCAGGTAAATAACGTTCAATTATTTATTGAACAATTT--------CTTATATTTAAGATTGTGCATAA

AAAATTAATTGAACTTAAGATTGATGAAATTCCTGCAATATGTAAGGAAAAAATTGTTAGATCAATAGAAGGATTTAATT

GAGTAGAAAGAGGAGGATAAATTGTTCATCCTGTTCCTGTTCCTGAACCAAAAATATTTCTGTAAATTAATAAGATTAAA

GAAGGAATTAATAATCAAAATCTTATATTATTTAATCGGGGAAATGCTATATCAGGGGCATTAATTATTAATGGAACTAA

TCAATTTCCAAATCCTCCAATTATGATTGGTATAACTATAAAAAAAATTATAATAAAAGCATGTGAAGTAACAATTGAAT

TATAAA-TTTGATCATTTCCAATTAGTATTCCTGGGATTCTTAATTC---------------
